# Single-cell transcriptome analysis reveals distinct cell populations in dorsal root ganglia and their potential roles in diabetic peripheral neuropathy

**DOI:** 10.1371/journal.pone.0306424

**Published:** 2024-07-31

**Authors:** Guojun Guo, Jing Chen, Qixiao Shen, Zhenbing Chen

**Affiliations:** 1 Department of Hand Surgery, Union Hospital, Tongji Medical College, Huazhong University of Science and Technology, Wuhan, Hubei, China; 2 Department of Dermatology, Traditional Chinese and Western Medicine Hospital of Wuhan, Tongji Medical College, Huazhong University of Science and Technology, Wuhan, Hubei, China; 3 Department of Orthopedics, Yangxin People’s Hospital, Huangshi, Hubei, China; ShanghaiTech University, CHINA

## Abstract

Diabetic peripheral neuropathy (DPN) is a common complication associated with diabetes, and can affect quality of life considerably. Dorsal root ganglion (DRG) plays an important role in the development of DPN. However, the relationship between DRG and the pathogenesis of DPN still lacks a thorough exploration. Besides, a more in-depth understanding of the cell type composition of DRG, and the roles of different cell types in mediating DPN are needed. Here we conducted single-cell RNA-seq (scRNA-seq) for DRG tissues isolated from healthy control and DPN rats. Our results demonstrated DRG includes eight cell-type populations (e.g., neurons, satellite glial cells (SGCs), Schwann cells (SCs), endothelial cells, fibroblasts). In the heterogeneity analyses of cells, six neuron sub-types, three SGC sub-types and three SC sub-types were identified, additionally, biological functions related to cell sub-types were further revealed. Cell communication analysis showed dynamic interactions between neurons, SGCs and SCs. We also found that the aberrantly expressed transcripts in sub-types of neurons, SGCs and SCs with DPN were associated with diabetic neuropathic pain, cell apoptosis, oxidative stress, etc. In conclusion, this study provides a systematic perspective of the cellular composition and interactions of DRG tissues, and suggests that neurons, SGCs and SCs play vital roles in the progression of DPN. Our data may provide a valuable resource for future studies regarding the pathophysiological effect of particular cell type in DPN.

## Introduction

Diabetic peripheral neuropathy (DPN) is the most common form of neuropathy, and have considerable morbidity, which occurs in approximately 50% of diabetic patients [1]. With the progress of the disease, the individuals may suffer from foot ulceration, neuropathic pain or even lower-limb amputation, and the quality of life is impaired significantly [2]. The pathogenesis of DPN is complex. Hyperglycemia, dyslipidemia and altered insulin signaling could cause various pathological alterations in peripheral nervous system, such as endoplasmic reticulum stress, DNA damage and mitochondrial dysfunction, and eventually result in DPN [3]. However, the distinct mechanisms of DPN development remain unclear.

Dorsal root ganglion (DRG) is situated between each spinal cord and spinal nerve on the posterior root, and is a critical structure in processing and transmitting the sensory neural signals from the periphery nerves to the central nervous system [4]. The DRG and peripheral nerve could undergo functional and structural damage caused by persistent diabetic status. Particularly, without the protection of the blood-nerve barrier, the DRG is a more vulnerable site compared with the peripheral nerve [5]. Animal models of DPN have revealed the pathophysiologic changes in DRG and their contributions to neuropathy [6]. Thus, the DRG tissue may be the primary choice in the research of DPN pathogenesis.

The pseudo unipolar cells within the DRG are somatosensory neurons, which enable body to detect and respond to various noxious and innocuous stimuli. In addition to sensory neurons, DRG also contains a variety of other cell types, such as satellite glial cells (SGCs), Schwann cells (SCs), fibroblasts, and immune cells [7]. Previous study has reported that the hypoxia-inducible factor-1 alpha (HIF-1α) signaling in diabetic peripheral sensory neurons is impaired, which has protective effect by suppressing nerve damage and promoting peripheral nerve survival [8]. The expression of lipocalin-2 (LCN2) is upregulated in SGCs from diabetic DRGs, subsequently promote the inflammatory responses in peripheral nervous system, and lead to DPN ultimately [5]. Besides, SCs apoptosis is occurred under high glucose, this pathological process is related with oxidative stress, autophagy, inflammatory reactions and so on, and indicate neuropathy [9]. Therefore, different cell types in DRG tissue could have distinct critical roles in the pathogenesis of DPN.

Single-cell RNA-seq (scRNA-seq) technology has been used extensively and rapidly in the biological areas in recent years. scRNA-seq can identify the cell types based on their global transcriptome patterns, as well as identify the disease-associated gene expression changes in each cell type, and dissect the mechanisms underlying a given disease [10]. With these advantages, the scRNA-seq technology has the potential to uncover transcriptome pattern of each cell in DRGs and their roles in the DPN development. Former study has investigated the DRG neuron changes and the development of mechanical allodynia using scRNA-seq technology [11]. However, the expression patterns of other cell types, the interactions of different cell types, and their contributions to DPN still lack systematic evaluation in the literature.

Here, the major goals of this work were to comprehend the cellular composition and interactions of DRG tissues, and to understand the cellular change in DPN. Therefore, we performed scRNA-seq in DRG tissues isolated from healthy control and DPN rats, to conduct expression profiling in each cell type. As a result, we classified cell populations in DRG tissue to eight cell types, including neurons, SGCs, SCs, endothelial cells, mural cells, fibroblasts, macrophages, and neutrophils. Moreover, neurons, SGCs and SCs were the three major cell types in DRG, and were classified to several sub-types. In particular, the expression patterns, the cellular interactions, and the pathophysiological effects to DPN of these important cell types were further analyzed and compared.

## Materials and methods

### Animals

Healthy male SD rats (8 weeks old, 180–220 g) were used in this study, and were purchased from Experimental Animal Center, Tongji Medical College, Huazhong University of Science and Technology, Wuhan, China. Rats were placed in separated cages with free access to water and food. The surrounding environment was kept at 22–24°C temperature, 40% humidity, and 12 hours dark and light cycle. Diabetes was induced after one week of adjustable feeding. This study was approved by the Animal Ethics Committee of Huazhong University of Science and Technology, and was performed based on the National Institute of Health Guidelines and Regulations.

### Induction of diabetes

All rats were split into diabetic and control groups randomly. Diabetes was induced as described previously [12]. Briefly, diabetic group was received the streptozotocin (STZ) through a single intraperitoneal (IP) injection at a dose of 65 mg/kg body weight after a 12-hour fast. STZ was freshly dissolved in citrate buffer (0.1 M, pH 4.4 at 4°C) before injection. The control group was injected with an equal volume of citrate buffer alone. Rats with blood glucose levels greater than 300 mg/dL (16.7 mM) were considered diabetic and selected in our study. Rats were left for 8 weeks after STZ injection to allow for the DPN development in diabetic rats.

### Measurements and tissues

The blood glucose, body weight, water intake, food intake, urine volume and withdrawal threshold were measured as previously described [12]. Following 8-week diabetic duration, rats were executed by cervical dislocation and decapitation under sodium pentobarbital anesthesia, and bilateral L3-L6 DRGs were dissected and collected, which were immediately used for the preparation of single cell suspension.

### Cell suspension preparation

Collected DRGs were minced into small pieces using micro-scissors. Subsequently, DRGs were digested at 37°C using the following two enzymes: 0.1% type I collagenase (Sigma, St Louis, MO) for 40 min, and 0.25% Trypsin-EDTA (Gibco, Grand Island, NY) for another 20 min. At the conclusion of the digestion, the DRGs were transferred to a complete medium (RPMI 1640 + 0.04% BSA), triturated using a fire-polished Pasteur pipette, and filtered through a 70-μm then 40-μm cell strainer (Falcon). The dissociated cells were collected by centrifugation at 1000 r/min for 7 min. Through using the MACS Dead Cell Removal Kit (130-090-101), the dead cells were removed, and the cell suspensions were obtained with high quality, which were used for single-cell sequencing immediately.

### Single-cell sequencing

Cell suspensions were loaded on a Chromium Controller (10× Genomics, GCG-SR-1) to form the gel beads-in-emulsion (GEMs). The Barcoded gel beads labeled single cell populations were transferred into a tube strip for reverse transcription. Single-cell RNA-seq libraries were constructed using the Chromium Single Cell 3’ Library & Single Cell 3ʹ v3 Gel Beads (Chromium, PN-1000075) according to the manufacturer’s protocols. Briefly, the cell suspensions were mixed with RT-PCR reagents and Barcoded gel beads, were added to a Chromium chip. Then, the Chromium chip was placed in a Chromium Controller. The GEM-RT-PCR was conducted on a PCR instrument (Bio-rad, MyCycler) using the following program: 45min at 53°C; 5min at 85°C; hold at 4°C. Barcoded cDNA was extracted from the partitioning Oil, and amplified using cDNA Amplification Reaction Mix. The 10× Chromium kit, which included reagents for fragmentation, ligation and sample index PCR, were used to generated sequencing libraries. The final libraries were sequenced on an Illumina Novaseq platform. The original Sequencing data were stored in NCBI database with GEO accession number GSE248328.

### Sequencing data processing

The Cell Ranger software (10× Genomics, version 3.1.0) was applied to demultiplex cellular barcodes, map reads to the transcriptome and genome by the STAR aligner, and down-sample reads to generate normalized aggregate data across samples as required, producing a matrix of gene counts versus cells. To remove possible multiple captures, dead cells and low-quality cells, the following criteria were applied: the number of expressed genes per cell (median ± 4×MAD), the unique molecular identifier (UMI) counts per cell (median ± 4×MAD), and proportion of mitochondrial gene counts (< 20%). Principal component analysis (PCA) was carried out to reduce the dimensionality, and the data were visualized in two dimensions through the t-distributed stochastic neighbor embedding (t-SNE) method. Batch effect was corrected by mutual nearest neighbor detection [13]. The R package SingleR was used to infer the origin of each single cells and identify their cell types independently [14].

### Identification of marker genes and differentially expressed genes

The marker genes of each cluster were identified using Seurat FindAllMarker function [15]. The expression levels of those marker genes in specific cluster were significantly higher compared with other clusters, and had potential ability to verify and define the cell type of each cluster. The marker genes were visualized using VlnPlot and FeaturePlot functions. Differentially expressed genes between DPN group and control group were identified using MAST test through the Seurat package [15]. Only genes that were expressed in at least 10% of cells in either of the two groups were used for differential expression analysis. Significantly aberrantly expressed genes were chosen only if *P*-value < 0.05 and foldchange > 1.5.

### GO enrichment and KEGG pathway enrichment analysis

Both differentially expressed genes or marker genes in different cell types or sub-types were subjected to Gene ontology (GO) analysis and Kyoto Encyclopedia of Genes and Genomes (KEGG) pathway analysis. GO enrichment and KEGG pathway enrichment analysis of DEGs or marker genes were respectively performed using R (version 4.0.3) based on the hypergeometric distribution. The quantification of gene set expressions was conducted using R package Quantitative Set Analysis of Gene Expression (QuSAGE) [16].

### Pseudotime trajectory analysis and cell communication analysis

Single-cell pseudotime trajectory analysis was conducted to reveal the evolution of different cell types using Monocle2 algorithm (version 2.4.0) [17]. Cell communications were analyzed on the basis of ligand-receptor interactions by using CellPhoneDB [18], to reveal the diversity, complexity and dynamics of intercellular communication in a wide range of biological processes.

## Results

### Identification of multiple cell types in DRGs

**Fig 1:**
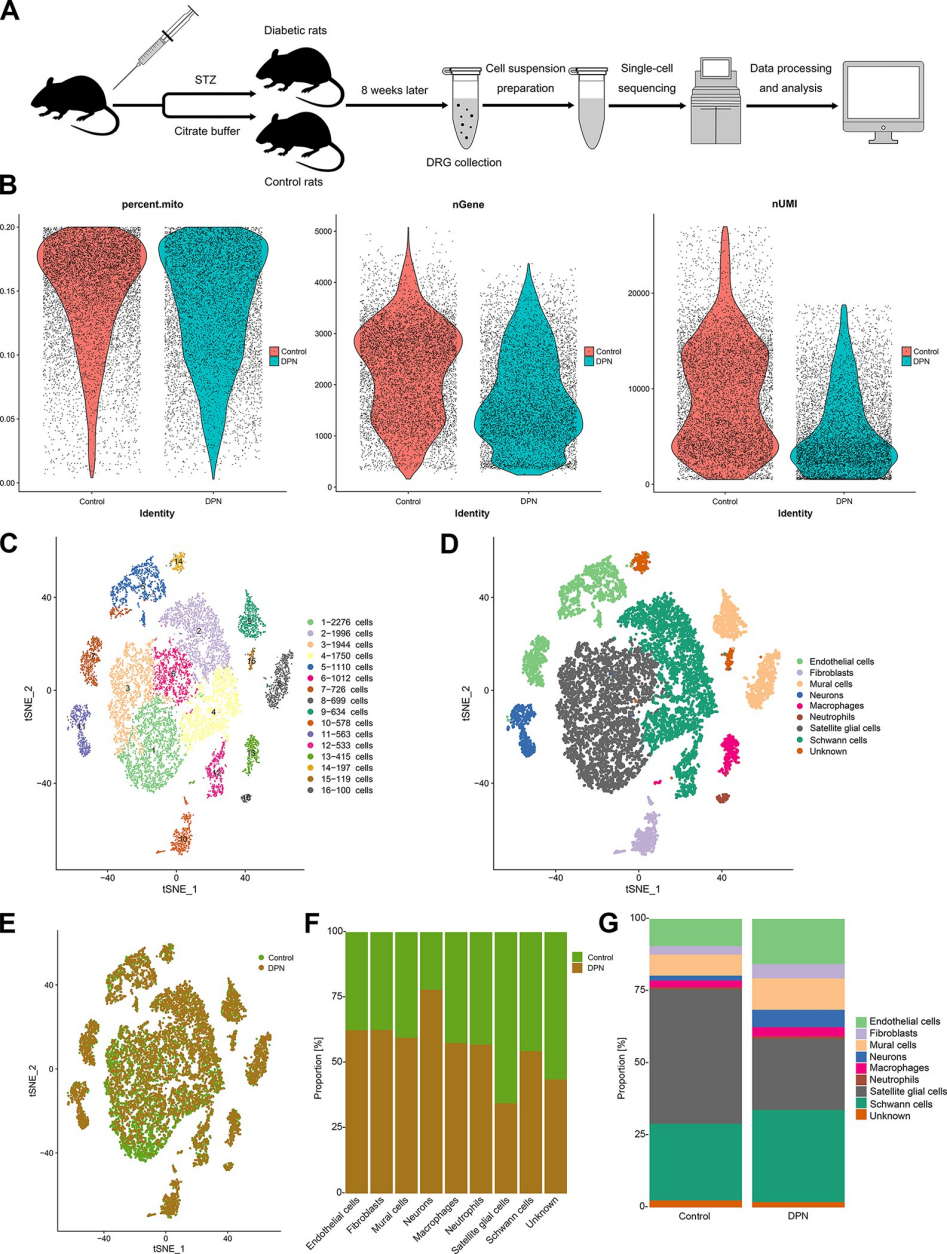
scRNA-seq identifies multiple cell types in DRGs. (A) Flow chart of DPN rat induction, sample collections and database construction. (B) Violin distribution map of the percentage of mitochondrial genes (percent.mito), the number of genes (nGene) and the number of UMIs (nUMI) detected in each cell after quality control. (C) A t-distributed stochastic neighbor embedding (t-SNE) plotting of the 14,652 cells showing 16 cell clusters. (D) A t-SNE plotting showing cell types for the 14,652 cells. (E) A t-SNE plotting showing cell populations colored as originating either from control tissues or from DPN tissues. (F, G) The proportion of each cell type in control and DPN tissues.

Based on the blood glucose, body weight, water intake, food intake, urine volume and withdrawal threshold, the STZ-injected rats developed DPN successfully (S1 Fig). The DRGs (bilateral L3-L6) from five control and five DPN rats were dissociated into single-cell suspensions, which were used to perform high-throughput scRNA-seq through 10× Genomics platform. Consequently, we obtained a total of 24,179 single cells, comprising 11,011 cells from the control group and 13,168 cells from the DPN group. After quality control (see filtering criteria in the methods), 14,652 single-cell transcriptomes (7,338 control and 7,314 diabetic) were retained for further analysis (Fig 1C). Following gene expression normalization, DRGs were classified into 16 clusters using FindVariableGenes function and FindAllMarkers function in Seurat package (Fig 1C). Each cluster included cells from both control and DPN DRG tissues (Fig 1E–1G).

**Fig 2:**
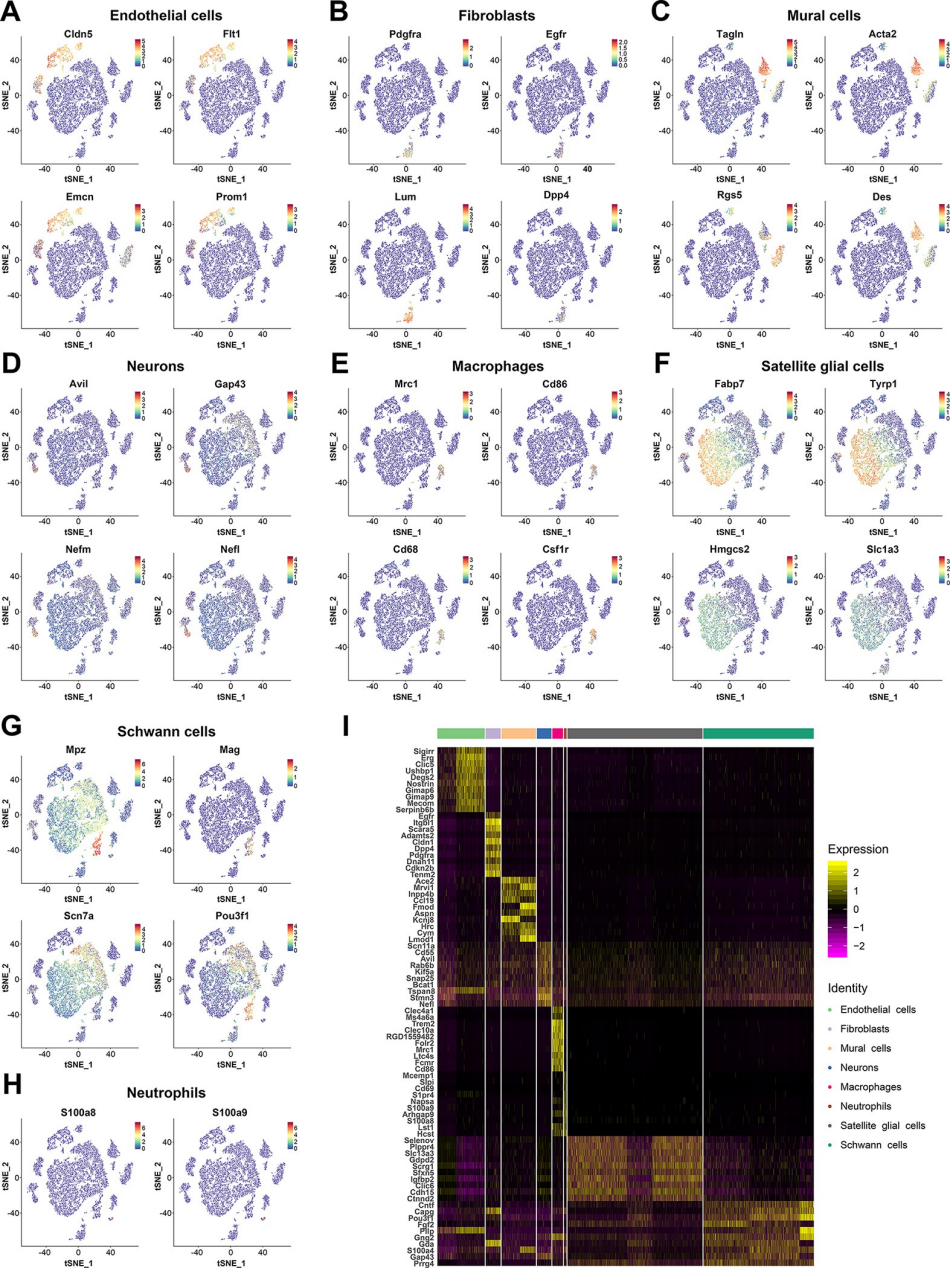
The marker gene identifies distinct cell types in DRGs. (A-H) The cell types identified by representative marker genes, using t-SNE plots. (I) Heatmap of expression signals of top marker genes in each cell type.

The transcriptionally distinct cell types were identified by using cell-type representative markers as well as cluster-specific genes (Fig 1D). Examination of the relative expression levels of marker genes in each cell type revealed eight cell types, including SGCs (Fabp7, Tyrp1, Hmgcs2 and Slc1a3) [7, 19], SCs (Mpz, Mag, Scn7a, Pou3f1) [20], neuron (Avil, Gap43, Nefl and Nefm) [21–23], endothelial cells (Cldn5, Flt1, Emcn and Prom1) [24, 25], mural cells (Tagln, Acta2, Rgs5 and Des) [26, 27], fibroblasts (Pdgfra, Egfr, Lum and Dpp4) [26, 28, 29], macrophages (Mrc1, Cd86, Cd68 and Csf1r) [30], and neutrophils (S100a8 and S100a9) [31]. We could not determine the cell types of clusters 14 and 15, and named them unknown cells. The distributions and expression levels of the representative marker genes in each cell types were shown in t-SNE plots (Fig 2A–2H), and the total gene list was shown in S1 Table.

### Identification of sub-types of neurons in DRGs

**Fig 3:**
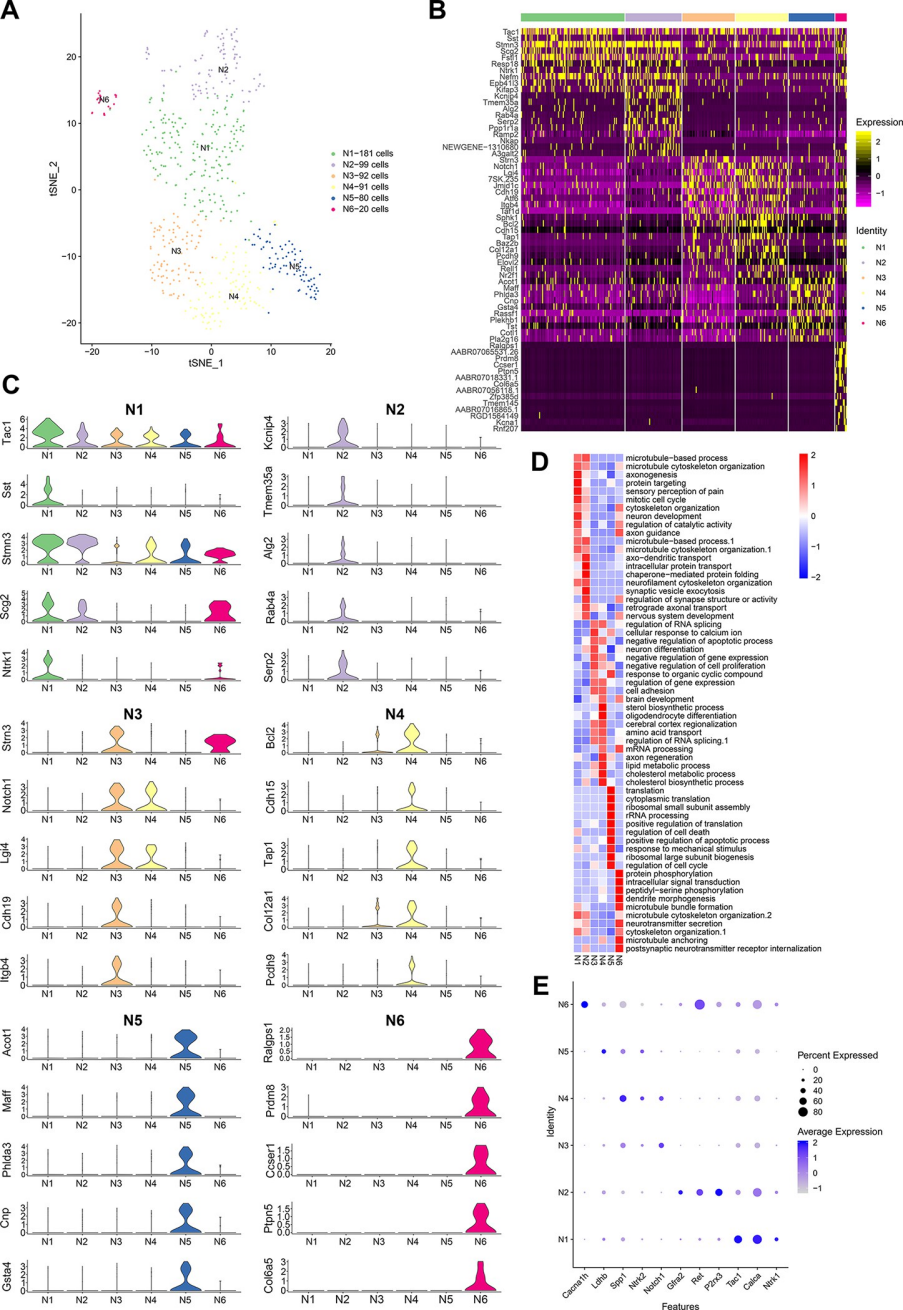
Top marker genes and biological processes in sub-types of neurons. (A) A t-SNE plotting showing six sub-types of neurons. (B) Heatmap of expression signals of top marker genes in each sub-type of neuron. (C) Violin plots showing the expression distribution for top marker genes in each sub-type of neuron. (D) GO analysis indicates enriched biological processes of each sub-type of neuron. (E) Bubble map showing the relative expression levels of reported marker genes in each neuron sub-type.

Cells in cluster 11 were interpreted as neurons (Fig 1C and 1D) and further sub-clustered into six groups by dimensionality reduction (Fig 3A). To identify the group-specific marker genes, the relative expression levels of genes in each group were also calculated. The distributions and expression levels of the top marker genes were displayed in violin plots (Fig 3C). Marker genes in neuron sub-type 1–6 (N1-N6) were illustrated in a heatmap (Fig 3B), and the gene list was shown in S2 Table.

According to previous studies, the N1 group could be classified as peptidergic (PEP) neurons, which expressed the classical markers such as neurotrophic receptor tyrosine kinase 1 (Ntrk1 or TrkA), calcitonin-related polypeptide (Calca or CGRP), and substance P (Tac1) [32]. And the N2 group might belong to the non-peptidergic (NP) neurons based on their highly expressed marker genes including P2rx3, Ret, and Gfra2 [32–34]. In addition, we concluded that the N4, N5 and N6 groups might be related to neurofilament containing (NF) neurons, which expressed the reported markers including neurotrophic receptor tyrosine kinase 2 (Ntrk2 or TrkB), secreted phosphoprotein 1 (Spp1), lactate dehydrogenase B (Ldhb), and calcium voltage-gated channel subunit alpha1 H (Cacna1h) [33] (Fig 3E). However, the N3 group did not match any reported sensory neuron cell types according to their expressed marker genes.

### Differentially expressed genes in neurons with DPN

**Fig 4:**
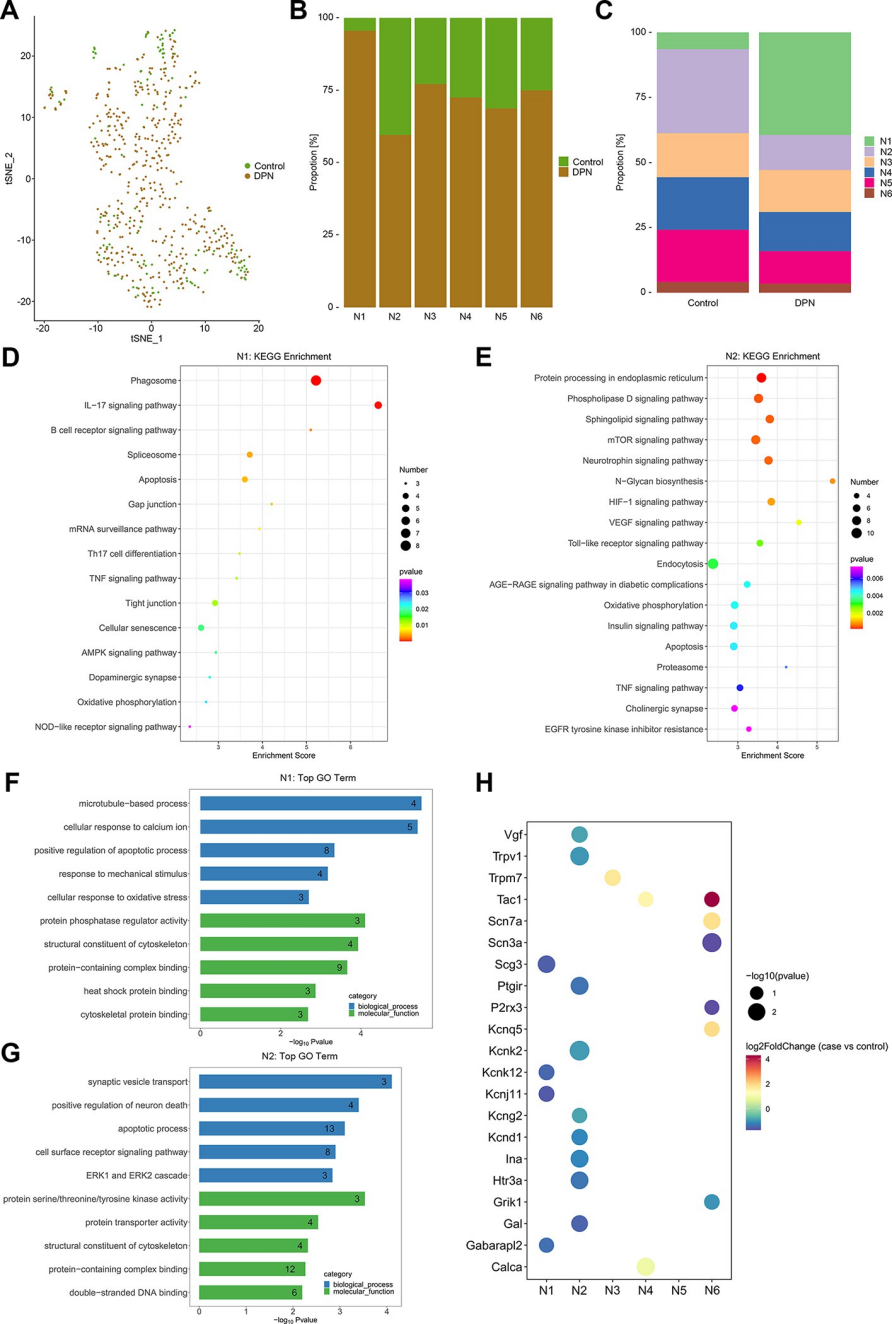
Aberrantly expressed genes and their biological functions in neurons with DPN. (A) A t-SNE plotting showing neurons colored as originating either from control tissues or from DPN tissues. (B, C) The proportion of each sub-type of neuron in control and DPN tissues. (D, E) KEGG pathways of aberrantly expressed genes in N1 and N2 neurons with DPN. (F, G) GO biological processes and molecular functions of aberrantly expressed genes in N1 and N2 neurons with DPN. (H) Bubble map showing aberrantly expressed genes in each sub-type of neuron with DPN.

By using the filtering criteria of *P*-value < 0.05 and foldchange > 1.5, the differentially expressed transcripts were identified in different neuron sub-types with DPN (S3 Table). All neurons from both groups were shown by the t-SNE dimension reduction approach (Fig 4A), and the proportion of each sub-type was shown in Fig 4B and 4C. Pathway and GO analyses of differentially expressed transcripts in each sub-type of neuron were shown in Fig 4D–4G, S2 and S3 Figs. Notedly, we found numerous voltage-gated, ligand-gated and transient receptor potential (TRP) channels were significantly differentially expressed across the neuronal sub-types, such as the voltage-gated sodium channels (including Scn3a and Scn7a in N6 group), the voltage-gated potassium channels (including Kcnk12 and Kcnj11 in N1 group, Kcnd1, Kcnk2 and Kcng2 in N2 group, Kcnq5 in N6 group), the ligand-gated ion channels (including Htr3a in N2 group, Grik1 and P2rx3 in N6 group), and the TRP channels (including Trpv1 in N2 group, Trpm7 in N3 group) [35]. Furthermore, lots of other operational components of sensory neurons were also aberrantly expressed, such as neurotransmission (including Scg3 in N1 group, Gal and Vgf in N2 group, Calca in N4 group, Tac1 in N4 and N6 groups), presynaptic regulation (Gabarapl2 in N1 group), chronic pain (Ptgir in N2 group), and conductive channel (Ina in N2 group) (Fig 4H) [33].

### Identification of sub-types of SGCs in DRGs

**Fig 5:**
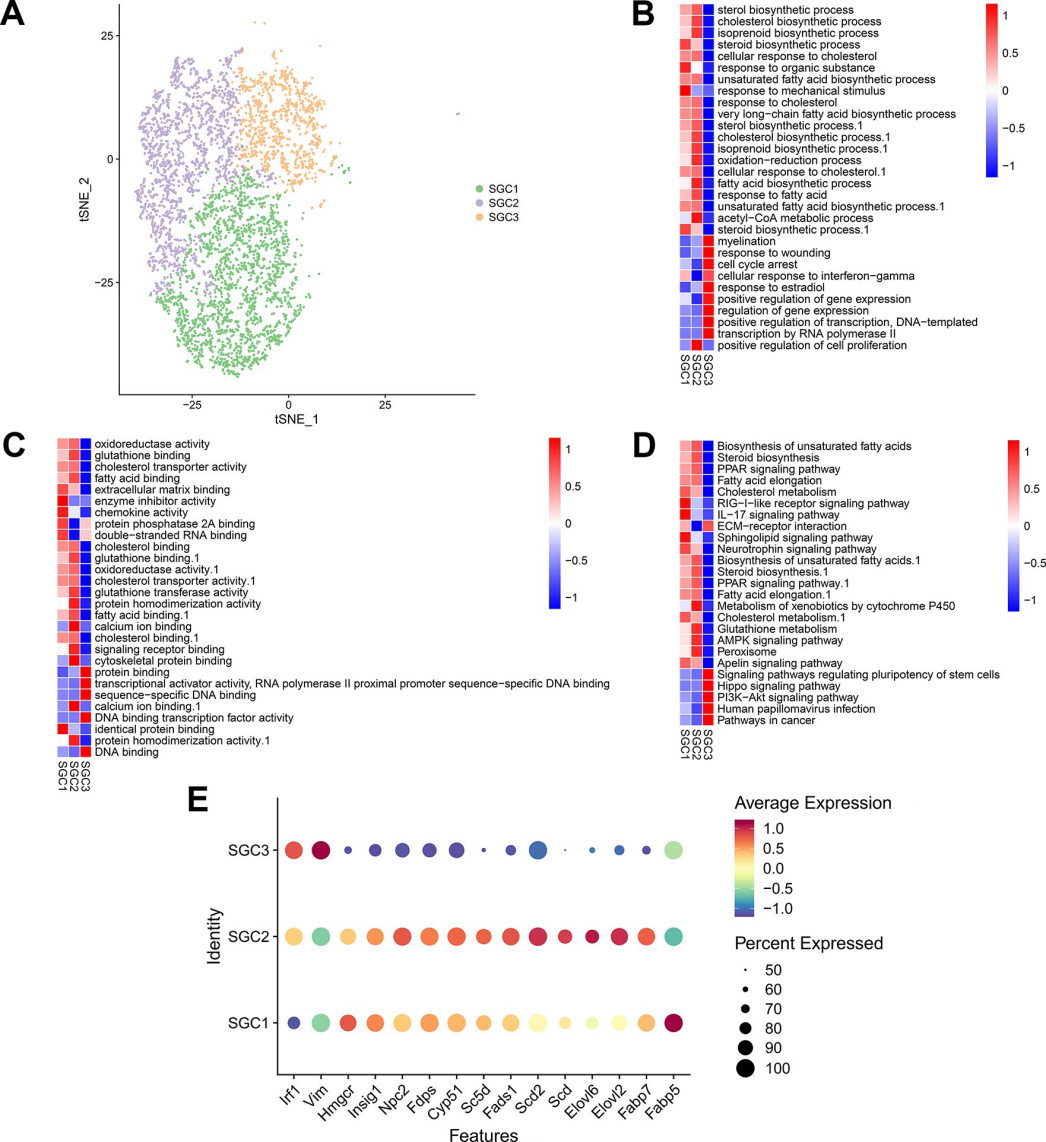
Identification of sub-types of SGCs in DRGs. (A) A t-SNE plotting showing 3 sub-types of SGCs. (B-D) Bioinformatics analyses indicates enriched biological processes (B), molecular functions (C) and pathways (D) of each sub-type of SGCs. (E) Bubble map showing the relative expression levels of reported marker genes in each SGC sub-type.

Based on the expression of known gene signatures, the cluster 1, 3 and 6 (also named as SGC1, SGC2 and SGC3, respectively) were classified as SGCs (Fabp7, Tyrp1, Hmgcs2 and Slc1a3) (Fig 1C and 1D). Marker genes in each SGC sub-type were shown in S4 Table. Particularly, the SGC1 and SGC2 groups shared plenty of common marker genes, and GO analysis indicated that those enriched genes were related with fatty acid metabolism, such as fatty acid binding proteins (Fabp5 and Fabp7), fatty acid elongases (Elovl2 and Elovl6), and desaturases (Scd, Scd2, Fads1 and Sc5d). Besides, the SGC1 and SGC2 groups also expressed marker genes associated with cholesterol metabolism, including Cyp51, Fdps, Npc2, Insig1, and Hmgcr [36]. In addition, we found the SGC3 group expressed a cohort of immune-related genes, such as vimentin (Vim) and interferon regulatory factor 1 (Irf1), indicating these cells might participate in defending DRGs against viral or bacterial infections [36, 37] (Fig 5B–5E).

### Identification of sub-types of SCs in DRGs

**Fig 6:**
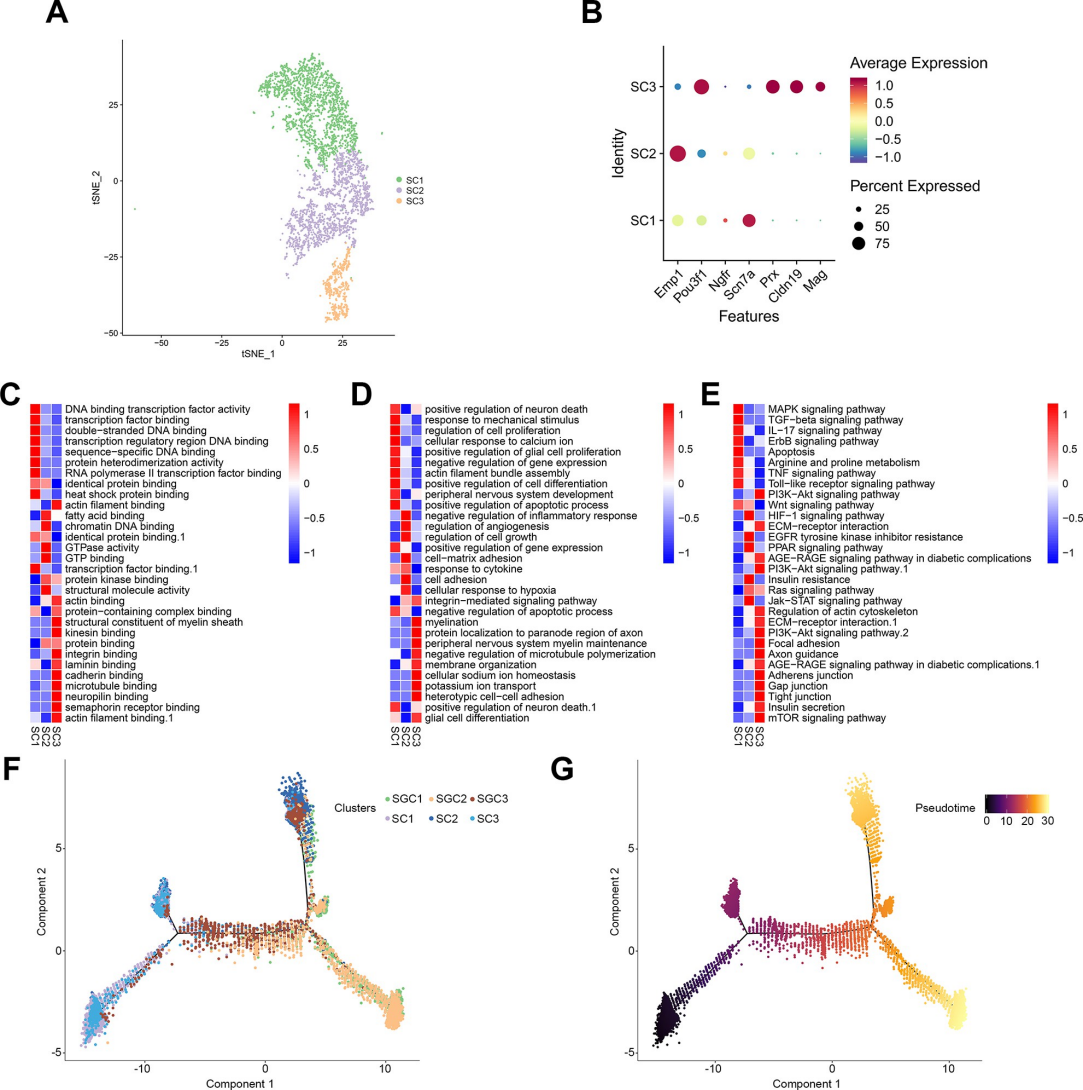
Identification of sub-types of SCs in DRGs and pseudotime trajectory analysis of peripheral glial cell sub-types. (A) A t-SNE plotting showing 3 sub-types of SCs. (B) Bubble map showing the relative expression levels of reported marker genes in each SC sub-type. (C-E) Bioinformatics analyses indicates enriched molecular functions (C), biological processes (D) and pathways (E) of each sub-type of SCs. (F, G) Inference of SGC and SC developmental connection by pseudotime trajectory analysis. SGCs and SCs exhibit distinct cell fates. Color key from bright to dark indicates cell progression from the early to the late stage.

By screening the top marker genes, we identified the cluster 2, 4 and 12 cells as SCs, each cluster represented one sub-types of SCs, and were named as SC1, SC2 and SC3, respectively. Marker genes in each SGC sub-type were shown in S5 Table. Among them, four top marker genes in SC3 were Mag, Cldn19, Pou3f1 and Prx, which were myelin-related proteins, and responsible for the process of myelination [20, 38]. Meanwhile, we found that the top transcript in SC1, Scn7a, belonged to the family of voltage-gated sodium channels to sustain the electrical activity in excitable tissues [20]. Besides, one top transcript in SC2, epithelial membrane protein (Emp1), belonged to the family of peripheral myelin protein 22 (PMP22), and participated in the neuronal differentiation and axon growth [39] (Fig 6B).

Considering the heterogeneity of sub-types in SCs, we performed pseudotime trajectory analysis of our single cell data on SCs using Monocle analysis [17]. As illustrated in the trajectory, SC1 and SC3 showed similar distributions along the pseudotime trajectory, primarily located on the left side of the trajectory; whereas SC2 was also distributed towards the upper right of the trajectory. Due to the relatively higher expression of myelin-associated genes in SC3, this suggested a higher degree of differentiation on the left side of the trajectory compared to the right (Fig 6F and 6G).

### The communication relationships between neurons, SGCs and SCs in DRGs

**Fig 7:**
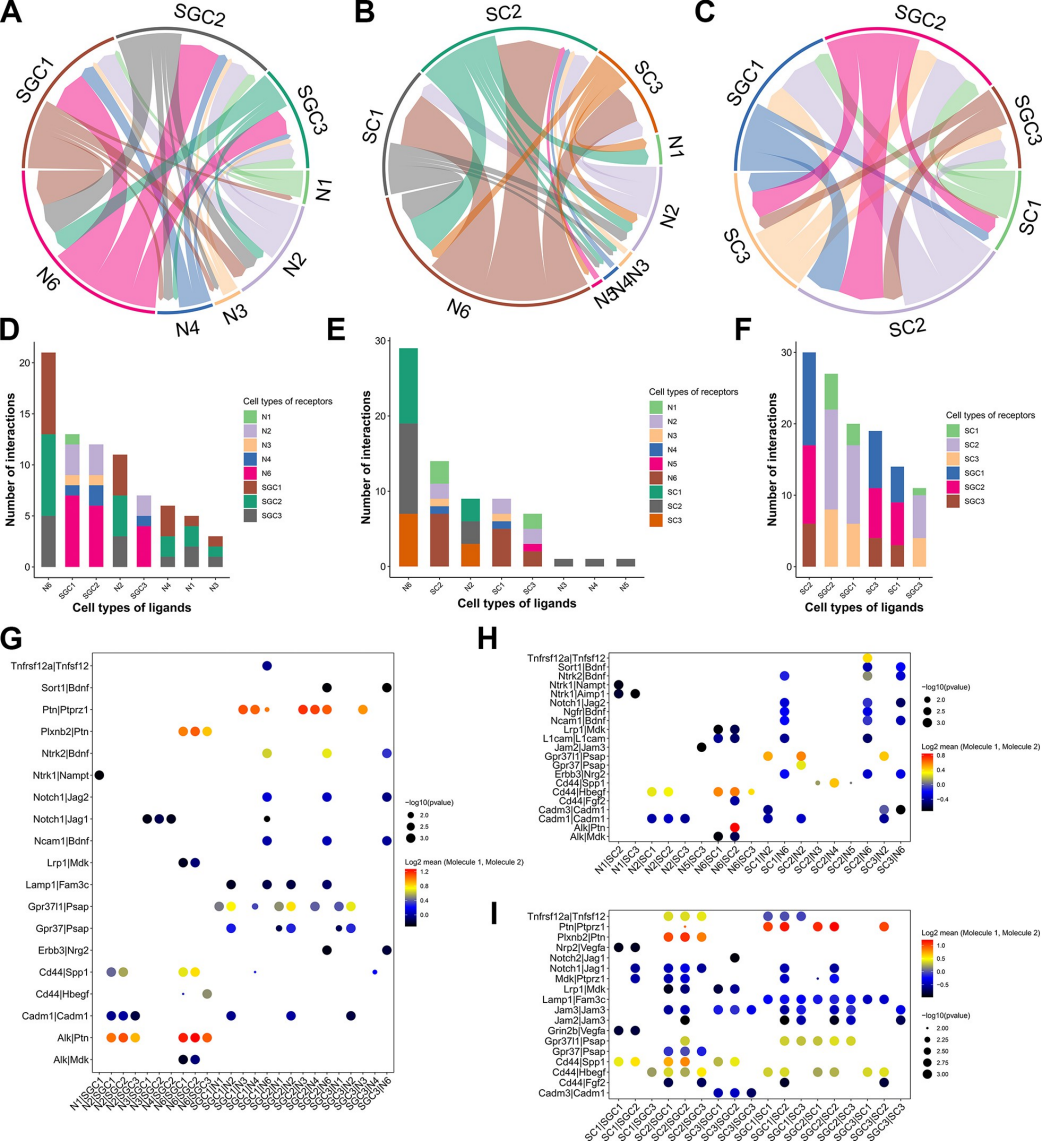
The cell-cell communications between neurons, SGCs and SCs in DRGs. (A-C) Chord diagrams showing the cellular communications between neurons, SGCs and SCs in DRGs. (D-F) Stacked bar graphs showing the number of interacting ligand-receptor pairs between neurons, SGCs and SCs in DRGs. (G-I) Ligand-receptor interactions between neurons, SGCs and SCs in DRGs.

The cellular communications between neurons, SGCs and SCs were evaluated by ligand-receptor pairs using CellPhoneDB [18] (Fig 7A–7C). We found that the strongest interaction between neurons and SGCs occurred between N6 and SGC1 or SGC2 (Fig 7D), interactions between neurons and SCs were higher between N6 and SC2 (Fig 7E), and interactions between SGCs and SCs were higher between SGC2 and SC2 (Fig 7F). Next, we further investigated the specific receptor-ligand pairs in different cell groups in detail. It was demonstrated that Alk on N6 closely bound to Ptn on SGC2 (Fig 7G) and SC2 (Fig 7H), and Ptn on SGC2 closely bound to Ptprz1 on SC2 (Fig 7I).

### Differentially expressed genes in SGCs with DPN

**Fig 8:**
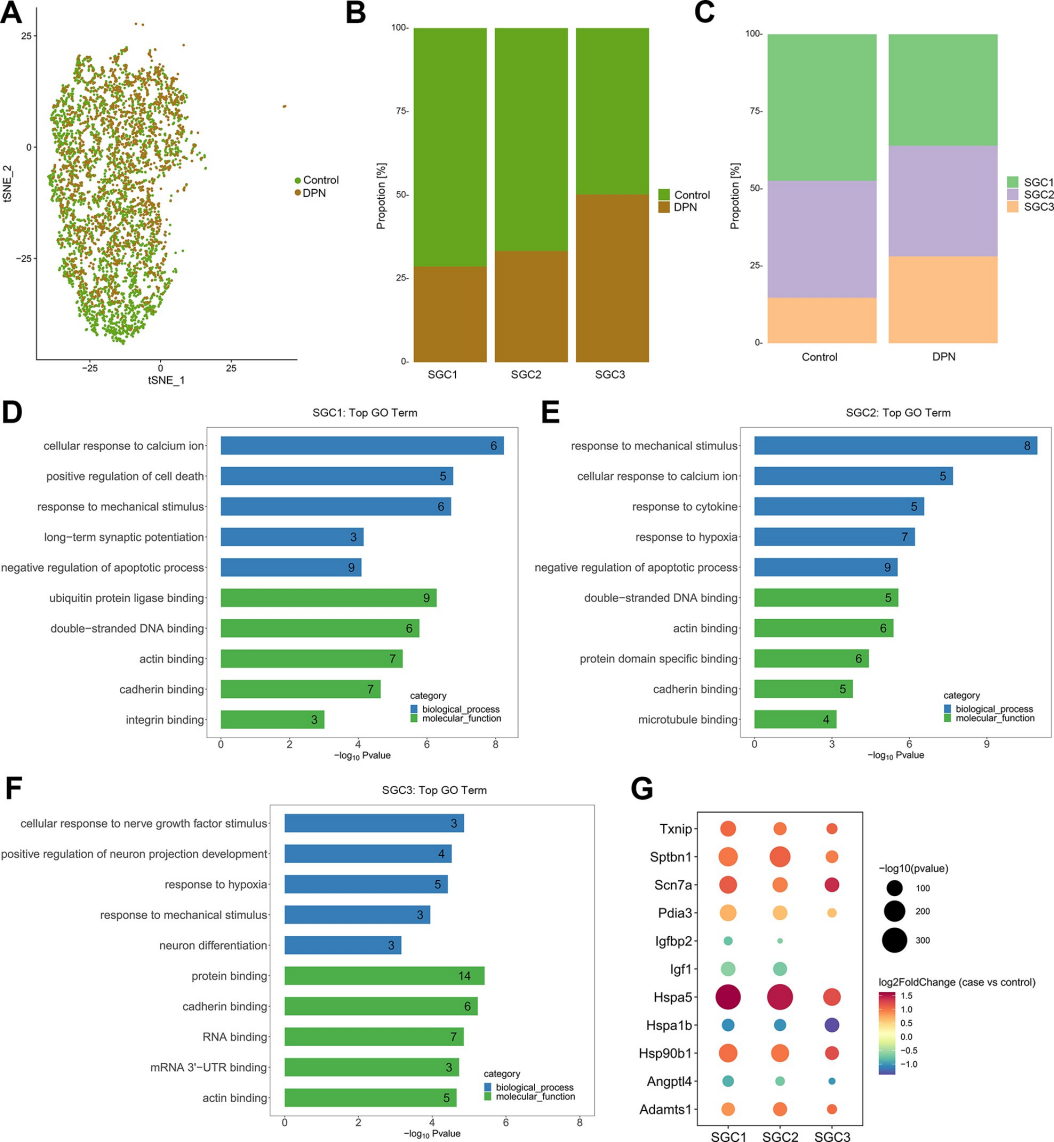
Aberrantly expressed genes and their biological functions in SGCs with DPN. (A) A t-SNE plotting showing SGCs colored as originating either from control tissues or from DPN tissues. (B, C) The proportion of each sub-type of SGCs in control and DPN tissues. (D-F) GO biological processes and molecular functions of aberrantly expressed genes in each sub-type of SGCs with DPN. (G) Bubble map showing aberrantly expressed genes in each sub-type of SGCs with DPN.

To isolate transcripts that were dysregulated in SGCs with DPN, differential expression analysis was conducted. As a result, all three cell groups of SGCs showed significantly differentially expressed genes with DPN (Fig 8 and S6 Table), and shared the most significantly downregulated genes, including Hspa1b and Angptl4. Interestingly, both these two genes were found to be involved in apoptotic process, which mediated anti-apoptotic effects to protect cells from multiple proapoptotic stimuli [40, 41]. Besides, growth factor related genes, Igf1 and Igfbp2, which were significantly downregulated in SGC1 and SGC2, were also apoptosis-associated genes, reported to have protective effects on cell growth and cell apoptosis [42, 43]. All SGC clusters also shared plenty of upregulated genes, such as heat shock protein genes (Hspa5 and Hsp90b1), which were known as unfolded protein response (UPR) genes, and could be activated by the endoplasmic reticulum (ER) stress [44]. Other mutual upregulated genes, such as Adamts1 and Pdia3, which were hypoxia-inducible gene [45] and proapoptotic response gene [46], respectively.

### Differentially expressed genes in SCs with DPN

**Fig 9:**
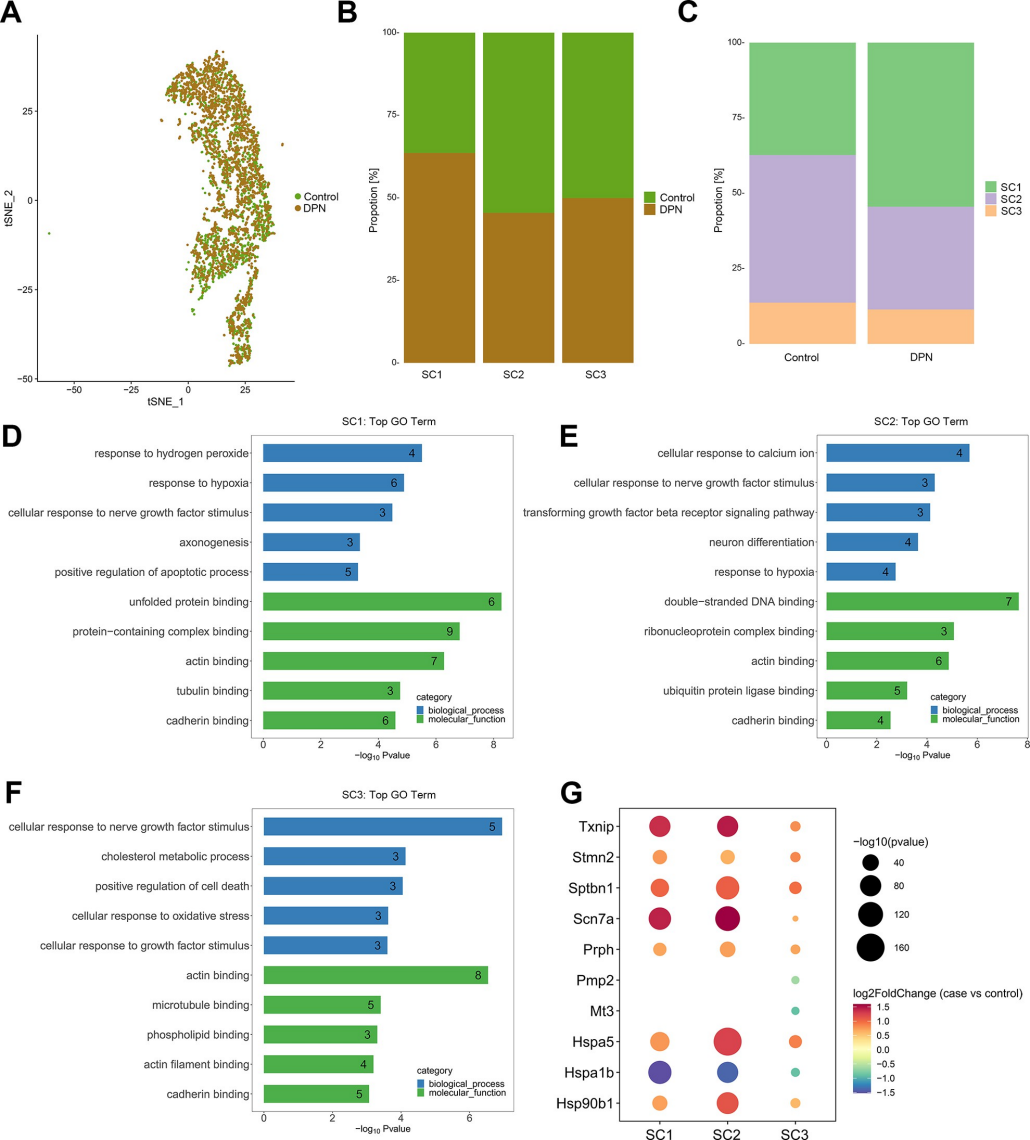
Aberrantly expressed genes and their biological functions in SCs with DPN. (A) A t-SNE plotting showing SCs colored as originating either from control tissues or from DPN tissues. (B, C) The proportion of each sub-type of SCs in control and DPN tissues. (D-F) GO biological processes and molecular functions of aberrantly expressed genes in each sub-type of SCs with DPN. (G) Bubble map showing aberrantly expressed genes in each sub-type of SCs with DPN.

Through differential expression analysis, lots of upregulated or downregulated transcripts in SCs with DPN were also identified (Fig 9 and S7 Table). We found the three clusters of SCs shared numerous dysregulated transcripts, and among them many transcripts were also aberrantly expressed in SGCs. For example, the upregulated transcripts including Scn7a, Txnip, Sptbn1, Hspa5 and Hsp90b1; and the downregulated transcripts including Hspa1b. The transcripts Scn7a played regulatory roles in diabetic neuropathic pain [47]; Txnip was the key factor to cause SC dysfunction in DPN [48]; Sptbn1 was involved in neurodegenerative diseases and exhibited roles in regulating axonal transport and neurite growth [49]; Hspa1b, Hspa5 and Hsp90b1 belonged to heat shock protein genes, Hspa1b had anti-apoptotic role to protect cells from multiple proapoptotic stimuli [40], and the latter two transcripts could be activated by the ER stress [44].

Additionally, the growth-associated marker genes, such as stathmin 2 (Stmn2) and peripherin (Prph), were upregulated in SC groups with DPN. Stmn2 and Prph were classical markers for axonal growth and neurite extension [50], indicating the SCs might exhibit some extent regeneration during the process of DPN. Besides, known genes associated with DPN were also identified in SCs, such as the downregulated genes metallothionein 3 (Mt3) and Pmp2 in mySC cluster [51, 52].

## Discussion

In the current study, by comprehensive analyzing single-cell sequencing data of DRG tissues in SD rats, we identified the complexity of cellular composition, and classified DRG cells into 8 main cell types, including neurons, SGCs, SCs, endothelial cells, mural cells, fibroblasts, macrophages, and neutrophils. Furthermore, our analysis revealed 6 sub-types of neurons, 3 sub-types of SGCs and 3 sub-types of SCs, provided the typical gene expression profile of each cell type. CellPhoneDB-predicted cell communications revealed close cell-cell interactions between neurons, SGCs and SCs. Besides, we analyzed the possible biological roles of neurons, SGCs and SCs in DPN. Our data showed dynamic gene expression alterations of these three cell types, which may play crucial roles in DPN.

The DRG neurons are somatosensory neurons, which serve to detect physical and noxious stimulation, and transmit these signals from peripheral nervous system into central nervous system. Particularly, previous study has dissected DRG neurons into four main types, including PEP, NP, NF and tyrosine hydroxylase containing (TH) neurons [33]. Consistent with former studies, we annotated the N1 cluster as PEP neurons, the N2 cluster as NP neurons, and the N4, N5 and N6 clusters as NF neurons based on the expression of known neuronal markers. Different neuronal types might have different functional assignments such as mechanosensitive, thermosensitive or nociceptive neurons [33]. We did not classify the N3 cluster as any known neuronal types, however, Notch1, one top marker gene in N3 group, was a neural progenitor proliferation marker [53], indicating this cluster might in the early stage of neuron development.

Interestingly, Zhou et al. also detected the sub-types of DRG neurons, and reclassified neurons according to the classical DRG neuron markers [11]. In accordance with our study, PEP neurons and NP neurons were identified. Based on the t-SNE plot, these two sub-types account for most of neurons. However, for the rest neuron sub-types, such as Trpm8-positive neurons (TRPM8), C-fiber low-threshold mechanoreceptors (C-LTMR) neurons or somatostatin-positive neurons (SOM), we could not identify them in our project. Particularly, the SOM neurons belong to NP3 neurons based on the study of Usoskin et al. [33]. Besides, the newly identified neurons by Zhou et al., MAAC neurons, could originate from PEP neurons on the basis of the gene expression profiles [11]. Future studies may combine these results to obtain a more complete classification of neurons.

We successfully induced the DPN rats using STZ injection [12], and identified the differentially expressed transcripts in neurons with DPN. Remarkably, lots of transcripts related to voltage-gated, ligand-gated and TRP channels were significantly aberrantly expressed. An increasing number of studies have reported various channels expressed in DRG neurons, which play vital roles in modulating the excitability of sensory neurons, and contribute to the development of painful symptoms directly [47, 54, 55]. Take the voltage-gated potassium channels for instance, our study identified multiple potassium channels which were downregulated in DPN, and the reduced expression of voltage-gated potassium channels in DRG neurons could increase the neuronal excitability and contribute to the diabetic neuropathic pain [54]. Together with other aberrantly expressed operational components known to participate in sensitization during neuropathic pain, which associated with neurotransmission, presynaptic regulation, chronic pain and conductive channel [33], we believed that the DRG neurons could play pivotal roles in the pathogenesis of diabetic neuropathic pain.

SGCs are flattened sheet-like cells, located in the surrounding of neuronal soma. In line with previous research, the SGCs we identified were enriched for genes involved in cholesterol biosynthesis and fatty acid metabolism (such as chaperone proteins, elongases and desaturases) [36], suggesting that lipid and cholesterol syntheses in SGCs are important to the associated neuronal compartments. A wide variety of neuronal stress situations, such as diabetes and traumatic nerve injury, could trigger the SGC activation, and activated SGCs are featured by profound changes [56]. Our differential expression analysis for SGCs revealed multiple dysregulated genes, such as apoptosis-associated genes, heat shock protein genes and hypoxia-inducible genes. Particularly, hypoxia is a significant etiologic factor in DPN, and numerous metabolic abnormalities, such as oxidative stress, could impair neural function and eventually cause cell apoptosis [57]. Besides, the aberrantly expressed heat shock protein genes are involved in ER stress, and dysfunction of ER affects lots of aspects of cell physiology and secretion, which ultimately leads to apoptosis of cells [44]. To sum up, those dysregulated genes reveal the possible ways of SGCs to participate in DPN.

Another glial cell type in DRGs are SCs, which wrap around axons in nerve trunks. SCs could be classified into myelinating cells and nonmyelinating cells on the basis of the way they interact with axons. In diabetic neuropathy, the myelinated and nonmyelinated axons were decreased, and the morphological changes and metabolic disorders were induced in SCs, such as aggregates of glycogen particles, edematous cell cytoplasm, activation of protein kinase C and polyol pathway hyperactivity [58]. Particularly, our single-cell sequencing data also identified numerous differentially expressed genes in SCs with DPN, which related to cell apoptosis, oxidative stress, diabetic neuropathic pain, SC dysfunction or demyelination. For instance, Mt3 and Pmp2 were downregulated in mySC cluster. As previously reported, metallothionein was a potent antioxidant to scavenge of free radicals, and oxidative stress was found to play vital roles in pathogenesis of DPN [51]; Pmp2 was expressed in myelin of peripheral nervous system which had essential roles in myelin sheath structure and nerve function, and mutant Pmp2 caused severe demyelination and decreased nerve conduction velocities [52]. Thus, the SCs could also play crucial role in the pathogenic mechanisms of DPN and need to be further studied.

Apart from the DPN model, the DRG tissues also exhibit cellular alterations in other injury models. Following peripheral nerve injury, scRNA-seq of the DRG tissues showed that multiple sub-types of the DRG neurons were in a regenerative condition [59], and the expression of classic regeneration-associated genes, such as Atf3 and c-Jun, were upregulated [60]. Besides, repair SCs were identified after spinal nerve transection, which specifically labeled by Shh, but the increase of repair SCs following sciatic nerve crush or transection were not detected [61]. Furthermore, following nerve injury, SGCs could promote axon regeneration of DRG neurons by upregulating genes related to the immune system and lipid metabolism [62]. However, pathological characteristics of DPN include nerve demyelination, axonal atrophy, cell apoptosis and delayed regeneration. Thus, the cellular alterations of DPN model are quite different from nerve injury models, and these differences may be instructive in the treatment of DPN.

As we known, neuronal cell bodies in DRG are covered SGCs, whereas axons in nerve trunks are ensheathed by nmSCs or mySCs. Cells in DRGs normally interact with other cells for cellular communications and signal transduction. For instance, neurons control SC functions by providing essential signals, whereas SCs promote neuronal survival and ensure efficient action potential transductions, and abnormal interactions of neuron-SC could result in diseases, including peripheral neuropathy [63]. Besides, SGCs in DRG participate in cellular communication through gap junctions, and the abnormal interactions between neurons and SGCs could contribute to various pain, such as post-herpetic pain, post-surgical pain, and diabetic neuropathic pain [64]. Also, SGC activation and TNF-α release could establish a neuron-glial communication in DRG, and play important roles in inflammatory visceral hyperalgesia [65]. Our bioinformatic results showed close cell-cell interactions among neurons, SGCs and SCs, and showed numerous specific ligand-receptor pairs on these cells. Notably, Alk is expressed in nociceptive DRG neurons and is involved in the neurons-SCs interaction [66], and Ptn is a secreted binding protein that exhibits vital roles in neural-glial interactions in the development of nervous system [67]. Thus, those ligand-receptor pairs might reveal specific ways of cell communication, which require further validation.

Although we identified multiple cell types in DRGs and investigated the roles of three major cell types in the pathophysiology of DPN, this study suffers from some limitations. For example, the diameters of some cells were too large to be captured by 10× Genomics, such as the large diameter neurons. In addition, some vulnerable cells may be loss during the digestion process. Thus, our data may not represent the actual cell proportion in DRGs. Furthermore, it was worth noting that signals from genes with high expression levels were analyzed while the low expression level genes might be neglected. Lastly, we did not discuss any cell types outside neurons, SGCs, and SCs, which may also play roles in DPN.

Despite these technical inherent defects and limitations, we reported the cellular and molecular landscape of DRG tissues at single-cell level. Our data revealed the complexity of cellular composition and dynamic gene expression alterations in DPN. These findings expand our understanding of the pathophysiological processes of DPN, and may serve as a resource for studying the functions of different cell types and treating DPN disease.

## Supporting information

S1 Fig The blood glucose, body weight, withdrawal threshold, water intake, food intake and urine volume of control rats and DPN rats. (A) The blood glucose levels were significantly greater in DPN rats compared with control rats. (B, C) The body weight and withdrawal threshold levels in DPN rats were significantly decreased. (D-F) The water intake, food intake and urine volume of DPN rats were significantly increased compared with controls. ***P < 0.001.
(TIF)

S2 Fig GO terms of aberrantly expressed genes in neuron sub-types. GO biological processes and molecular functions of aberrantly expressed genes in N3, N4, N5 and N6 neurons with DPN.
(TIF)

S3 Fig KEGG pathways of aberrantly expressed genes in neuron sub-types. KEGG pathways of aberrantly expressed genes in N3, N4, N5 and N6 neurons with DPN.
(TIF)

S1 Table Marker genes of eight cell types in DRG. The list of marker genes of eight cell types in DRG.
(XLSX)

S2 Table Marker genes of neuron sub-types in DRG. The list of marker genes of neuron sub-types in DRG.
(XLSX)

S3 Table Differentially expressed genes in neuron sub-types with DPN. The list of Differentially expressed genes in neuron sub-types with DPN.
(XLSX)

S4 Table Marker genes of SGC sub-types in DRG. The list of marker genes of SGC sub-types in DRG.
(XLSX)

S5 Table Marker genes of SC sub-types in DRG. The list of marker genes of SC sub-types in DRG.
(XLSX)

S6 Table Differentially expressed genes in SGC sub-types with DPN. The list of differentially expressed genes in SGC sub-types with DPN.
(XLSX)

S7 Table Differentially expressed genes in SC sub-types with DPN. The list of differentially expressed genes in SC sub-types with DPN.
(XLSX)
