# Supplementary figures and images for: Single-cell transcriptome analysis reveals distinct cell populations in dorsal root ganglia and their potential roles in diabetic peripheral neuropathy
Source: PLoS One. 2024 Jul 31;19(7):e0306424. doi: 10.1371/journal.pone.0306424 (PMC11290642; doi:10.1371/journal.pone.0306424)

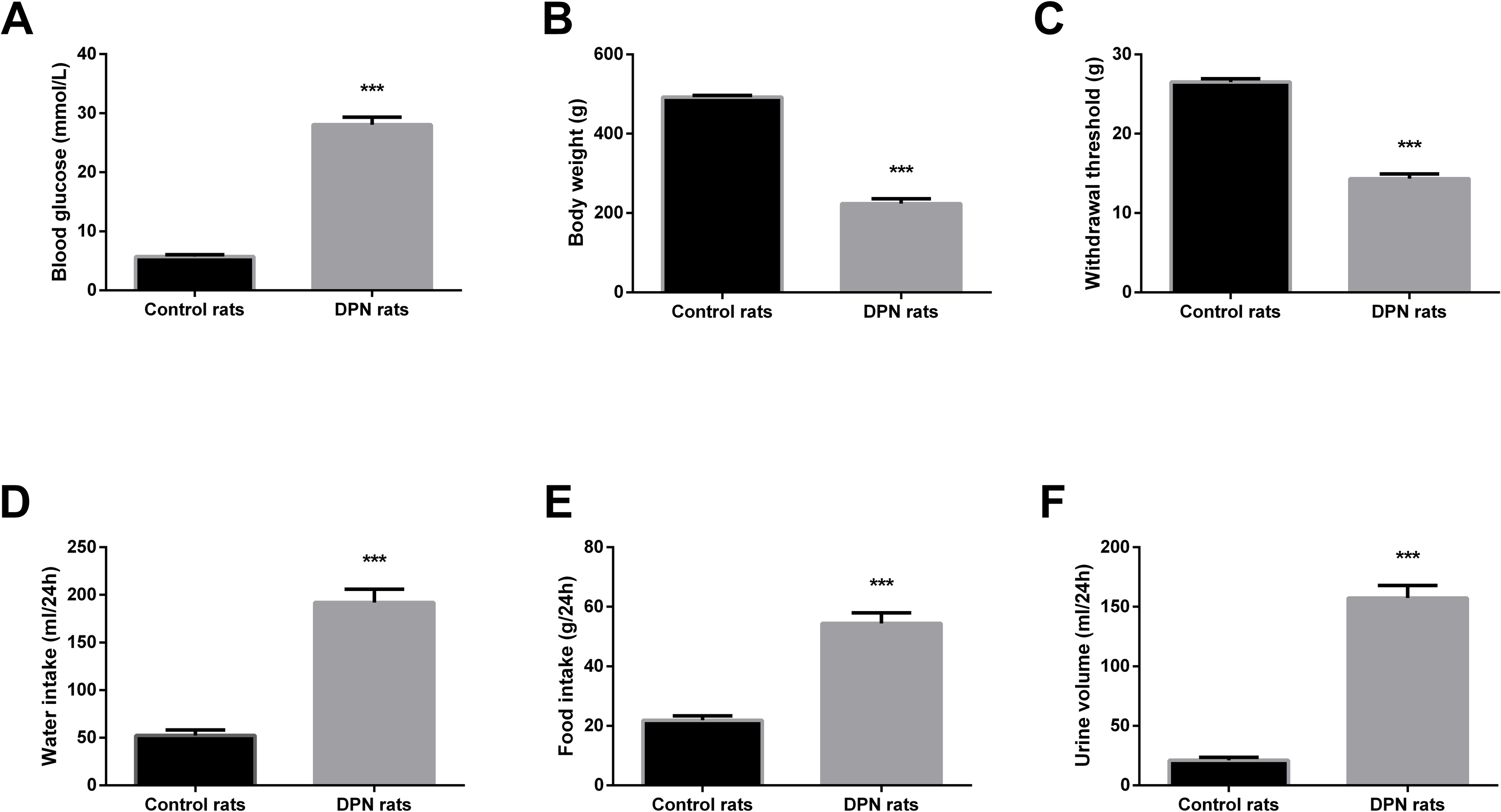

Supplement: S1 Fig — (A) The blood glucose levels were significantly greater in DPN rats compared with control rats. (B, C) The body weight and withdrawal threshold levels in DPN rats were significantly decreased. (D-F) The water intake, food intake and urine volume of DPN rats were significantly increased compared with controls. ***P < 0.001. (TIF) [file pone.0306424.s001.tif]

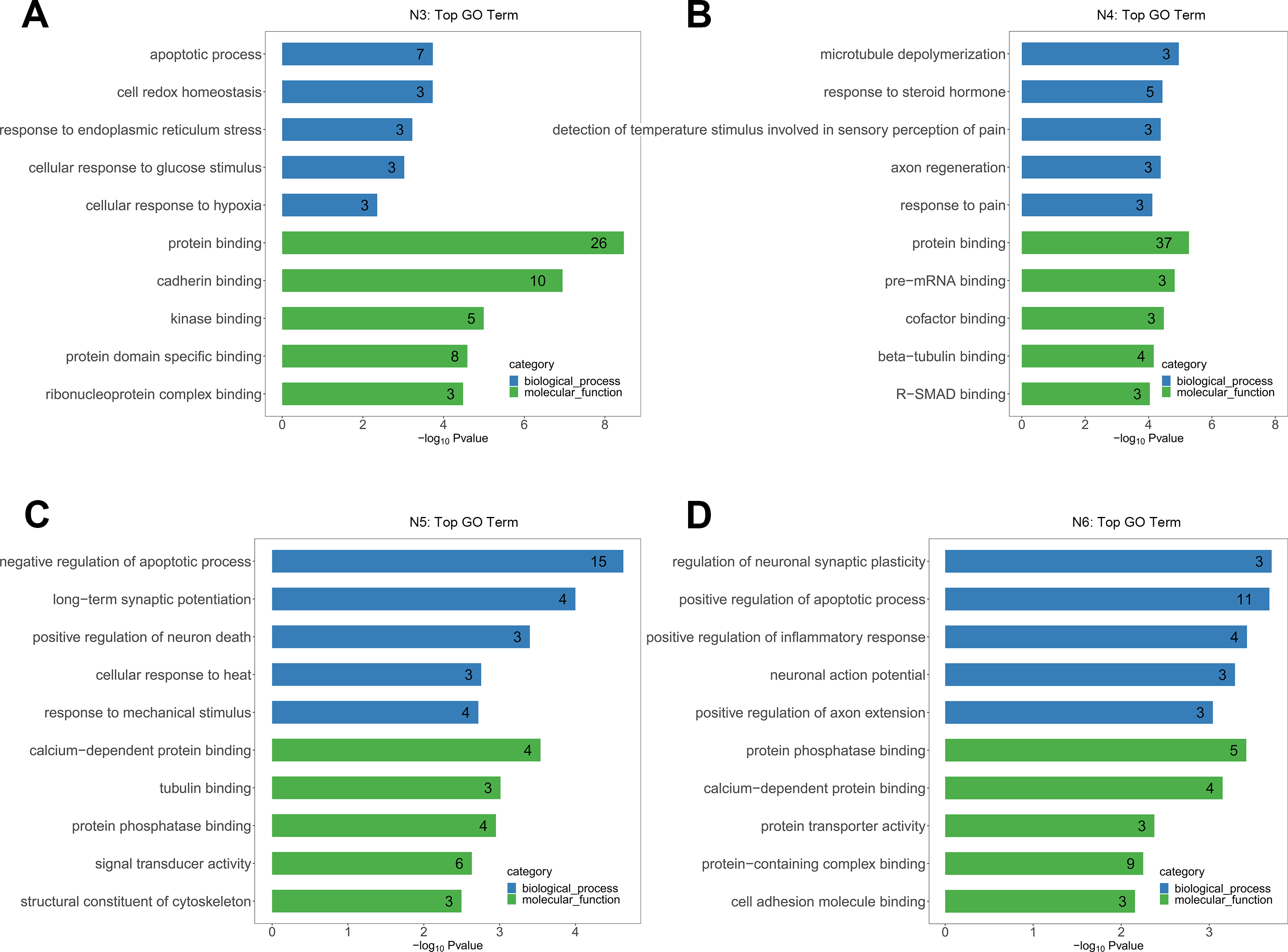

Supplement: S2 Fig — GO biological processes and molecular functions of aberrantly expressed genes in N3, N4, N5 and N6 neurons with DPN. (TIF) [file pone.0306424.s002.tif]

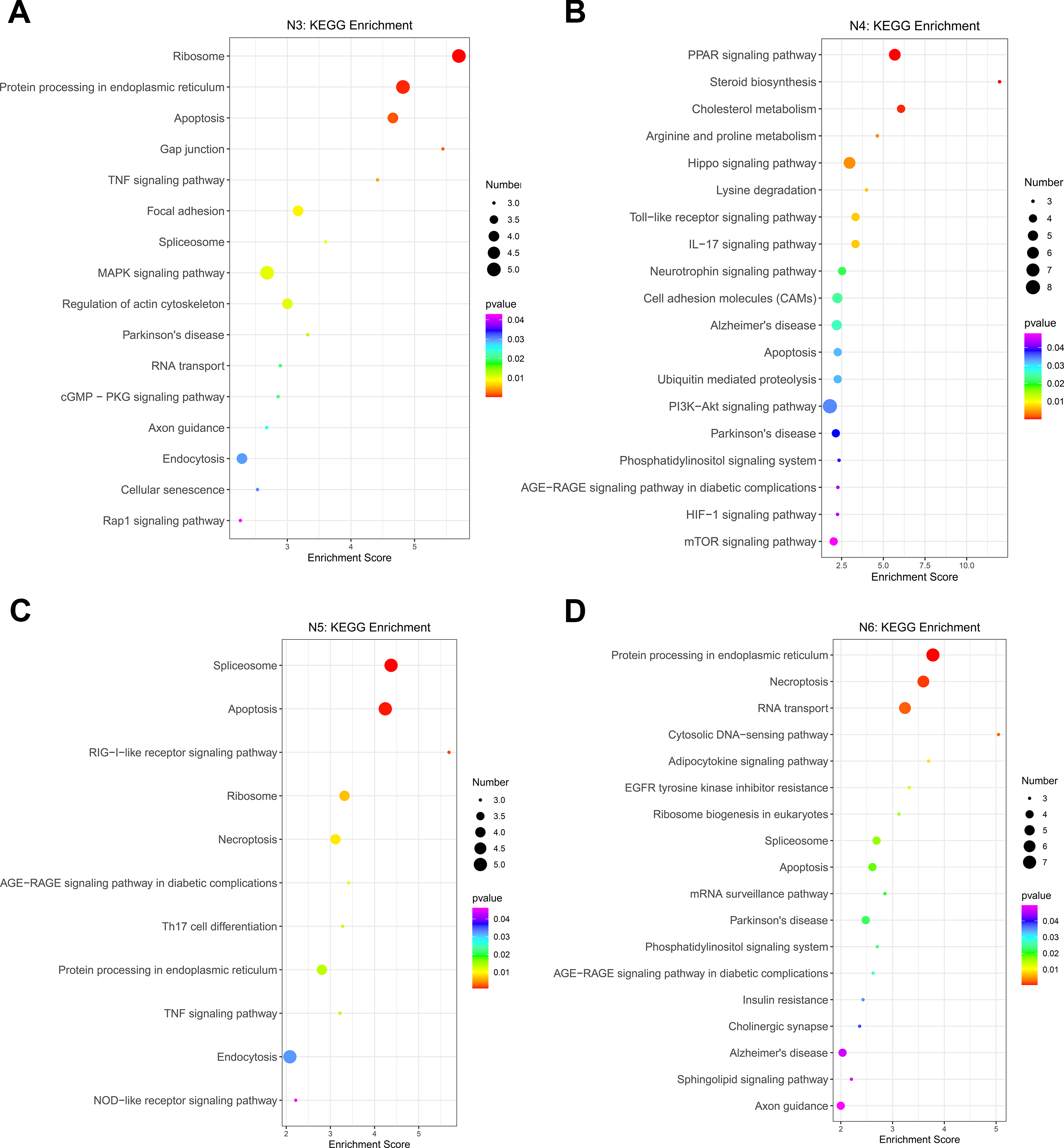

Supplement: S3 Fig — KEGG pathways of aberrantly expressed genes in N3, N4, N5 and N6 neurons with DPN. (TIF) [file pone.0306424.s003.tif]
